# Let-7b-mediated pro-survival of transplanted mesenchymal stem cells for cardiac regeneration

**DOI:** 10.1186/s13287-015-0221-z

**Published:** 2015-11-05

**Authors:** Jie Cheng, Ping Zhang, Hongbing Jiang

**Affiliations:** Jiangsu Key Laboratory of Oral Diseases, Nanjing Medical University, 136 Hanzhong Road, Nanjing, 210029 Jiangsu Province China

## Abstract

Stem cell-based repair and regeneration for cardiac regeneration following myocardial injury remain unmet challenges largely due to low viability of cells transplanted in the recipient sites. Accumulating evidence has revealed that local existence of reactive oxygen species (ROS) causes transplanted cell death via both apoptosis and autophagy. Ham and colleagues have identified let-7b as one of the primary mediators for ROS-induced apoptosis and autophagy of mesenchymal stem cells (MSCs) through direct targeting of caspase-3. Importantly, intramyocardial injection of let-7b-modified MSCs significantly enhanced ventricular function and facilitated myocardial repair by protecting transplanted cells from apoptosis and autophagy in the rat cardiac ischemia-reperfusion model. These findings provide novel insights into the roles of microRNA underlying stem cell survival following in vivo delivery, and offer further evidence that microRNA-modified MSC transplantation might be an effective therapeutic approach for tissue repair and regeneration.

## Introduction

Ischemic heart disease, one of the most common causes of mortality worldwide, is characterized by myocardial injury caused by oxygen deprivation which results in cardiomyocyte death and impaired cardiac functions and, if not properly managed, associates with mortality and morbidity. In past decades, stem cell-based regenerative medicine has revolutionized biomedical science and it holds extraordinary translational potential as exemplified by the fact that mesenchymal stem cells (MSCs) are an attractive source for cell-based regenerative therapies in ischemic myocardial injury on the basis of their multi-potent differentiation potential and pro-angiogenic as well as immunomodulatory properties. However, one of the main obstacles for successful stem cell-based regeneration is the low cellular viability following transplantation, which remarkably compromises therapeutic efficacy and outcomes. So there is a critical and urgent need for the development of an optimized strategy to promote cell survival in the recipient organs. Recently in *Stem Cell Research & Therapy*, Ham and colleagues [1] reported that the microRNA let-7b can potently improve the survival of MSCs by inhibiting apoptosis-related caspase-3 and enhance ventricular function, microvessel density, and recovery of ischemic myocardial injury. These findings further support the key roles of microRNA in cardiac repair and regeneration and provide novel evidence that microRNA-modified MSC delivery might be a novel and viable treatment strategy for ischemic heart disease.

## MicroRNA-mediated MSC therapy in myocardial injury

Ischemic injury results in metabolic and structural changes in affected cells because of deprivation of oxygen and nutrition, inevitably leading to various types of cell death such as apoptosis or autophagy. Various surgical and pharmacological approaches have been routinely used to treat this disease in the clinic. However, such approaches cannot efficiently save the cardiomyocytes and restore the cardiac functions and this is largely due to the limited regenerative potential of cardiac progenitors following injury. The advent of MSC identification and functional characterization has offered another possibility to treat ischemic cardiac injury. Indeed, MSC transplantation for this disease has been shown to be highly beneficial and valuable to repair or regenerate novel cardiomyocytes in the infarcted regions of the heart in multiple animal models and selected clinical patients. However, the harsh microenvironment in the damaged heart significantly undermines the survival of transplanted MSCs, thus greatly hampering their therapeutic efficacy and outcomes [2]. Therefore, optimizing the approaches to augment engrafted cell survival and function is a prerequisite to translate this therapeutic strategy into clinics. Until now, several approaches have been proposed to promote cell survival, including additions of growth factors, gene or microRNA modifications, and chemically activating signaling pathways in MSCs before transplantation [3–5]. In particular, the microRNA molecules, a novel class of endogenous post-transcriptional regulators, stand out given their well-established roles underlying cardiac repair [6]. Moreover, these single-stranded RNAs have been shown to hold tremendous potential as therapeutic targets and this is largely due to the regulatory models of microRNA such as simultaneous multiple targeting and modest regulation of target gene expression [7]. Ham and colleagues and others have shown that microRNA-modified MSCs were more conducive and effective to repair of infarct injury and improved heart function by enhancing transplanted cell survival and cardiomyogenic differentiation [1, 2], thus highlighting the benefits and potential of microRNA-modified MSCs in the treatment of ischemic heart disease.

One of the important findings from the study by Ham and colleagues is that the let-7b suppresses autophagic activity in MSCs as shown by the fact that autophagy-related genes *Atg5*, *7*, and *12* and *beclin-1* were significantly downregulated in let-7b-overexpressed MSCs. Indeed, mounting evidence indicates that autophagy has a double-edged role in balancing cell survival and death. It occurs primarily as the last survival mechanism to promote cell longevity under conditions of cellular stress and is not directly responsible for cell death. Under conditions of nutrient deprivation, hypoxia, or inflammation, autophagic degradation is one of the main strategies not only to eliminate unwanted cytoplasmic materials but also to recycle and subsequently re-use intracellular components such as amino acids to promote cell survival. Interestingly, autophagy can also trigger cell death, termed autophagic cell death, which has been called non-apoptotic programmed cell death [8]. Although Ham and colleagues reported that autophagic activity and the expression of autophagy-related genes were attenuated upon exogenous let-7b introduced into MSCs, it is still unclear how let-7b regulates autophagy and whether autophagy attenuated by let-7b is directly associated with MSC in vivo survival. Therefore, it will be an interesting question for further study to unravel the complex crosstalk among let-7b, cellular apoptosis, and autophagy in MSCs under specific conditions.

Notably, the study by Ham and colleagues has revealed that delivery of let-7b-overexpressed MSCs markedly promotes neovascularization in the infarcted myocardium and functional recovery of damaged heart as assessed by significantly increased ejection fraction and cardiac output as well as approximately 10 % reduction of fibrotic area in the let-7b-overexpressed MSC delivery group as compared with control. Although self-renewal and multi-differentiation potential of MSCs might be responsible for the improved cardiac repair, there is still controversy about the precise mechanisms underlying this process. Some researchers argue that MSCs can differentiate into cardiomyocyte-like cells within distinguishable morphology and similar functions as compared with native cardiomyocytes, but others argue that these beneficial effects might be due to the paracrine signals or factors secreted from MSCs. For example, MSC-mediated tissue protection and repair are partially achieved through the release of growth factors such as vascular endothelial growth factor and basic fibroblast growth factor to promote angiogenesis. In addition, MSCs are able to create an immunosuppressive microenvironment to alleviate local inflammatory responses after transplantation [9]. In the study by Ham and colleagues, the transient survival of MSCs through DAPI (4′,6-diamidino-2-phenylindole) staining at 3 days after transplantation was assessed. However, this seems relatively short, and in vivo long-term cellular lineage tracking might offer more compelling evidence to support the conclusion.

MSC-based therapy appears as a powerful therapeutic approach in ischemic myocardial injury. However, more studies are still needed to demonstrate MSCs as the optimal cells to undergo cardiomyogenesis and sustain long-time improvement of heart function. Recent study has proposed a novel approach by using cell fusion to support myocardial regeneration. Cardiochimeras formed by fusion between cardiac-derived progenitor cells and bone marrow-derived MSCs have enhanced reparative potential in a mouse model of myocardial infarction. Moreover, cardiac-derived progenitor cells are capable of undergoing direct cardiomyogenic differentiation, whereas MSCs provide protective immunomodulatory function and growth factor release in a paracrine manner [10].

## Conclusions

Ham and colleagues have provided evidence that let-7b might promote cellular survival of MSCs both in vitro and in vivo presumably by preventing cell apoptosis and autophagy, thus enhancing cell survival in the treatment of ischemic heart disease. It is worth mentioning that research on the efficacy and safety of MSC-based therapy in human cardiac repair is now under way. Although many challenges lie ahead, we believe that MSC-based therapy will be a sound approach for myocardial injury in the near future.

## Abbreviations

MSC: mesenchymal stem cell
ROS: reactive oxygen species
